# Monitoring and analysis of the change process in curriculum mapping compared to the National Competency-based Learning Objective Catalogue for Undergraduate Medical Education (NKLM) at four medical faculties. Part II: Key factors for motivating the faculty during the process

**DOI:** 10.3205/zma001083

**Published:** 2017-02-15

**Authors:** Maria Lammerding-Koeppel, Marianne Giesler, Maryna Gornostayeva, Elisabeth Narciss, Annette Wosnik, Stephan Zipfel, Jan Griewatz, Olaf Fritze

**Affiliations:** 1 University of Tuebingen, Faculty of Medicine, Competence Centre for University Teaching in Medicine Baden-Wuerttemberg, Tuebingen, Germany; 2 University of Freiburg, Medical Faculty, Competency Centre for Evaluation in Medicine Baden-Wuerttemberg, Freiburg, Germany; 3 University of Heidelberg, Medical Faculty, Center of Excellence for Assessment in Medicine - Baden-Wuerttemberg, Heidelberg, Germany; 4 University of Heidelberg, Medical Faculty Mannheim, Competence Centre of Final Year, Mannheim, Germany; 5 University of Tuebingen, Faculty of Medicine, Dean's Office of Student Affairs, Tuebingen, Germany

**Keywords:** Curriculum mapping, competence orientation, competency-based, medical education, change management, motivation

## Abstract

**Objective: **After adoption of the National Competency-based Learning Objectives Catalogue in Medicine [Nationaler Kompetenzbasierter Lernzielkatalog Medizin, NKLM], the German medical faculties are asked to test the learning obejctives recorded in it and evaluate them critically. The faculties require curricular transparency for competence-oriented transition of present curricula, which is best achieved by systematic curriculum mapping in comparison to the NKLM. Based on this inventory, curricula can be further developed target-oriented.

Considerable resistance has to be expected when a complex existing curriculum is to be mapped for the first time and a faculty must be convinced of its usefulness. Headed by Tübingen, the faculties of Freiburg, Heidelberg, Mannheim and Tübingen rose to this task. This two-part article analyses and summarises how NKLM curriculum mapping was successful at the locations despite resistance. Part I presented the resources and structures that supported implementation. Part II focuses on factors that motivate individuals and groups of persons to cooperate in the faculties.

**Method: **Both parts used the same method. In short, the joint project was systematically planned following the steps of project and change management and adjusted in the course of the process. From the beginning of the project, a Grounded-Theory approach was used to systematically collect detailed information on measures and developments at the faculties, to continually analyse them and to draw final conclusions.

**Results:** At all sites, faculties, teachers, students and administrative staff were not per se willing to deal with the NKLM and its contents, and even less to map their present curricula. Analysis of the development reflected a number of factors that had either a negative effect on the willingness to cooperate when missing, or a positive one when present. These were: clear top-down and bottom-up management; continuous information of the faculty; user-oriented support in the mapping process by reduction of the mapping categories, portioning and condensation of the NKLM via student pre-mapping (blueprint) and visibility of growing consent. Apart from that, there were a series of frequent questions, objections and concerns that could be countered strategically and by argumentation. They particularly referred to relevance, benefit, feasibility and effort of curriculum mapping.

**Conclusion:** An overview of beneficial framework conditions, strategies and results from different points of view is achieved and interrelations are made visible. Based on literature results, the motivating factors as well as their implementation and effects in the faculties involved are critically reflected on. Recommendations can be derived that can support other faculties in practice.

## Zusammenfassung

**Fragestellung: **Mit Verabschiedung des Nationalen Kompetenzbasierten Lernzielkatalogs Medizin [NKLM] sind die medizinischen Fakultäten gefordert, die hierin erfassten Lernziele zu erproben und kritisch zu evaluieren. Für den kompetenzorientierten Umbau bestehender Curricula benötigen die Fakultäten curriculare Transparenz, die am besten durch ein systematisches Curriculummapping im Abgleich zum NKLM erreicht wird. Auf Basis dieser Bestandsaufnahme können die Curricula gezielt weiterentwickelt werden.

Erfahrungsgemäß ist erheblicher Widerstand zu erwarten, wenn ein komplexes, bereits bestehendes Curriculum erstmals kartiert werden soll und eine Fakultät von dem Nutzen überzeugt werden muss. Unter Tübinger Leitung stellten sich die Fakultäten Freiburg, Heidelberg, Mannheim und Tübingen dieser Aufgabe. Der vorliegende zweiteilige Artikel analysiert und bilanziert die Erfahrungen, wie trotz Widerständen das NKLM-Curriculummapping in den Standorten gelungen ist. In Teil I wurden die für die Implementierung förderlichen Ressourcen und Strukturen dargestellt. Der vorliegende Teil II fokussiert auf die motivierenden Faktoren, die die Bereitschaft zur Mitarbeit in den relevanten Personengruppen der Fakultäten beeinflussen.

**Methodik: **Das methodische Vorgehen war in Teil I und II des Artikels identisch. Das Verbundprojekt wurde im Vorfeld systematisch nach Schritten des Projekt- und Changemangements geplant und im Projektverlauf angepasst. In einem Grounded Theory-Ansatz wurden von Anfang an detaillierte Informationen zu Maßnahmen und Entwicklungen an den Fakultäten über verschiedene Quellen und Methoden systematisch zusammengetragen, laufend analysiert und abschließend bilanziert.

**Ergebnisse: **An allen beteiligten Standorten waren Fakultätsleitungen und -mitglieder, Lehrende, Studierende und administrative Mitarbeiter nicht per se bereit, sich mit dem NKLM als solchem und über die Kartierung mit dem bestehenden Curriculum auseinanderzusetzen. Die Analyse der Entwicklungen ergab einerseits eine Reihe von Faktoren, die sich bei Fehlen ungünstig auf die Bereitschaft zur Mitarbeit auswirkten bzw. diese begünstigten, wenn sie vorhanden waren. Diese waren: Klare Top-down- und Bottom-up-Führung; kontinuierliche Information der Fakultät; bedarfsgerechte Unterstützung im Mapping-Prozess durch Reduktion der Mapping-Kategorien, Portionierung und Kondensierung des NKLM über studentisches Vormapping (Blueprint) sowie Sichtbarkeit der wachsenden Zustimmung. Andererseits gab es zusätzlich eine Reihe von häufigen Fragen, Einwänden und Befürchtungen, denen strategisch und argumentativ erfolgreich entgegengetreten werden konnte. Sie betrafen insbesondere Relevanz, Nutzen, Machbarkeit und Aufwand des Curriculummappings.

**Schlussfolgerung:** Aus verschiedenen Blickwinkeln wird ein Überblick zu förderlichen Rahmenbedingungen, Strategien und Ergebnissen erreicht und Zusammenhänge sichtbar gemacht. Die motivierenden Faktoren sowie ihre Umsetzung und Wirkungen in den beteiligten Fakultäten werden auf der Basis von Literaturergebnissen kritisch reflektiert. Daraus lassen sich Empfehlungen ableiten, die anderen Fakultäten Hilfestellung für die Praxis geben können.

## 1. Introduction

### Background: Challenges to NKLM curriculum mapping

In accordance with international developments [4], the National Competency-based Learning Objective Catalogue Medicine [NKLM, retrieved on 22.03.2016] was adopted in Germany by the Medical Faculties Conference (oMFT [Ordentlicher Medizinischer Fakultätentag]) in June of 2015 [3]. The comprehensive catalogue will initially have the status of recommendation for restructuring the study of medicine until 2020 and should now be tested by the faculties. After a revision, it should apply to all faculties.

Most faculties educated for some years with reformed study plans in which present NKLM content, like promotion of practical skills and communication abilities was already integrated [23]. In curricula that have grown over time, new and old content and examinations are often not efficiently linked and systematically structured [2], [8]. The transition to competence orientation requires effective change management that again achieves the necessary curricular transparency best by systematic curriculum mapping [2], [10], [15], [24]. A curricular map that displays important components of the curriculum and its links in the sense of “what, when, how, by whom” in comparison with the NKLM [8], enables departments to communicate about the curriculum for targeted further development [19]. For all involved and particularly for lecturers, NKLM curriculum mapping is a laborious measure and its benefits initially hard to understand. Consequently, there is a lot of resistance to it [11]. That is also borne out by the experiences at four faculties in Baden-Württemberg. Led by the faculty of Tübingen, the faculties of Freiburg, Heidelberg, Mannheim and Tübingen implemented curriculum mapping against the NKLM [http://www.merlin-bw.de/, retrieved on 15.05.2016].

In Part I of this article it has already been shown which resources and structures should be made available to prepare the mapping process: trained coordinators, systematic project management, organisation and communication structures, web based mapping instrument (MER*LIN* database [5]) and others. Part II focuses on which key factors could be identified for motivating deans (of study and teaching) administrative staff, students and particularly lecturers to cooperate, and how frequent problems can be dealt with strategically and argumentatively.

## 2. Methods

The methodical approach was identical in Part I and Part II. Therefore, only a brief overview is given, whilst reference is made to Part I for the details.

### 2.1. Database

The report covers the activities of four medical faculties in Baden-Württemberg during the period from 2012 (project start) until 2015 (end of the first mapping round). The initial basis was the NKLM version of February 2013, from June 2015 onward, the final version formed the basis.

#### 2.2. Project planning and management

The change process as well as organisation and communication structures have been systematically planned and adjusted to needs during the process. In workshops and regular, semi-annual project meetings, strategies and measures have been jointly further developed and coordinated cross-locational and within the faculties, their implementation at the locations has been discussed and the results have been reflected upon. All workshops and project meetings have been carefully logged and documented in terms of aims, progression and results and commented upon in memos.

#### 2.3. Data acquisition

Based on a Grounded-Theory approach [22], detailed information on structures and developments at the faculties has been gathered since the beginning of the project through various sources and methods and has been evaluated in parallel. In conclusion, correlations and conjunctions have been identified and recommendations for practical use have been derived. All gathered data has been anonymised, so that no persons or locations were recognisable.

## 3. Results

At all locations similar experiences were gained: Decision makers and administrative staff in dean’s offices and departments and particularly lecturers were not *per se* ready and willing to deal with the NKLM and its contents concretely and even less to conduct curriculum mappings. Initially, the lacking discernment in the necessity, especially among lecturers, often led to insufficient acceptance and disregard of information, to critical but polite rejection, up to open resistance.

The analysis of the change process revealed several relevant factors that facilitated readiness to cooperate, resp. had unfavourable impact in the case of failures: clear top-down and bottom-up management; continuous information to the faculty; adjusted support during the mapping process; visibility of the growing approval. Besides, there were a number of frequently asked questions, complaints and fears that could be successfully countered strategically and argumentatively. They related particularly to relevance, benefit, feasibility and effort of the curriculum mapping. In the following, they will be dealt with one by one.

### 3.1. Relevant factors, enhancing the willingness to cooperate

**Clear guidance.** The mapping process was very slow to get of the ground, if the dean and/or the dean of study and teaching or another authorised person in an accepted position did not officially support the project and when required, paved the way for the project staff in faculty and committees. Concrete and helpful top-down measures were, for instance: official support letter from the dean to the department heads; explicit empowerment of the staff member; support for project presentations in committees; (also brief) public statements with endorsement of the project and its necessity; if required, critical reflection on the project from a fundamentally positive perspective. On locations where such support was missing, project staff made only very slow progress with the mapping, despite putting in great efforts.

The involvement of an experienced bottom-up manager was also highly relevant. Particularly at the beginning of the project, fundamental conviction, strong communication skills, endurance, frustrations tolerance, structuring skills and personal continuity were required. The knowledge of departments “open to innovations”, good horizontal networking throughout the faculty and effective collaboration with the dean of study and teaching and the management of the dean’s office of student affairs improved acceptance and support and thereby the likelihood of presentable early successes.

**Continuous communication with the faculty. **In the relevant committees and target groups it became apparent that broad and continuous information and (also personal) persuasion in favour of the NKLM and the status of the mapping project brought about greater familiarity with the project and more participation. At one location, the multi-layered, regular information activities included for example: Meetings with the dean of study and teaching, study commission, faculty management, closed meetings on teaching, closed meeting of the chair holders, day of teaching, meetings with the teaching and examination coordinators, semester representatives, students, newsletters, posters, teacher trainings, etc. At locations, where project staff had no access or only limited access to official committees or where the NKLM curriculum mapping and its value were not an issue in public and committees, the project progress was delayed substantially.

**Adjusted support for lecturers in the mapping process.** It was indispensable at the locations that, on the one hand, lecturers required sufficient knowledge as operative basis for mapping to understand the background and significance of the task, and on the other hand, could be sure to get all the necessary support when it was needed. The following measures, offered at two levels, have proved themselves: workshops and individual counselling.

Short workshops (of two to four hours), in which not only knowledge was imparted but particularly mapping was done together, were considered to be recommendable by the coordinators because of their time efficiency and networking possibilities: Lecturers from various disciplines, working together in interdisciplinary courses or (sequentially) teaching similar contents/competencies, benefited from close exchange and mutual assistance.

When required, (additional) one-on-one advice/guidance was provided by the coordinator or trained (student) staff members, which, although time-consuming, was worthwhile in the end. Representatives of disciplines, who wanted to do the mapping alone from the beginning or who left the one-on-one session to continue working alone, were frequently incapable to complete the task because of the still on-going daily obligations. Suitable, constant reminding contact as well as the possibly renewed offer of one-on-one support by the coordinator, was crucial in that case.

**Visibility of the approval. **When a “critical mass” [11], [12] had been achieved at a faculty, statements appeared simultaneously from different sources, such as “There is no real resistance any more at our location”, “Mapping is no longer being questioned.” On the way, measures could be identified that made the growing acceptance visible. In workshops with teaching coordinators, it could be observed that critics and proponents were often in balance. Arguments of the approving peers were often more readily accepted than arguments from project staff. Supporting lecturers were asked to take position on their experiences in committees. By publicly presenting the disciplines who had already completed the mapping, the process developed its own dynamism. Generally, it was very beneficial to identify the proponents amongst the teaching coordinators at an early stage and to start the mapping process with these persons. In that way, trainings could be optimised and better attuned to the requirements of the relevant target group. The mapping process became more effective with that approach. Following the official adoption of the NKLM, almost everywhere a noticeable boost toward more readiness to implement curriculum mapping could be noticed.

#### 3.2. Dealing with relevant questions and complaints during the process 

Repeated analyses of strengths and weaknesses during the process made it possible for the project staff to find location-specific strategies and to argue depending on the location. Questions about relevance, benefit, feasibility and required effort were of major interest. It was crucial to have convincing arguments and examples ready at the right time. Understanding the profit clearly reduced the resistance.

**Relevance.** According to recorded notes, particularly at management levels, the clear political will was seen as an important argument against opposing subject-related and individual interests. The approval of the NKLM once again clearly increased the significance of the mapping. The fact that the learning objective catalogue is a recommendation until 2020 and not mandatory, temporarily provoked a wait-and-see attitude in many departments and faculty managements or minimal engagement (“Sit it out?”). In committee discussions, it was seen as an advantage to use the coming five years as a competitive edge for the location to position itself on the basis of its local experiences and to participate in shaping the prospective NKLM evidence-based.

**Figure 1:**
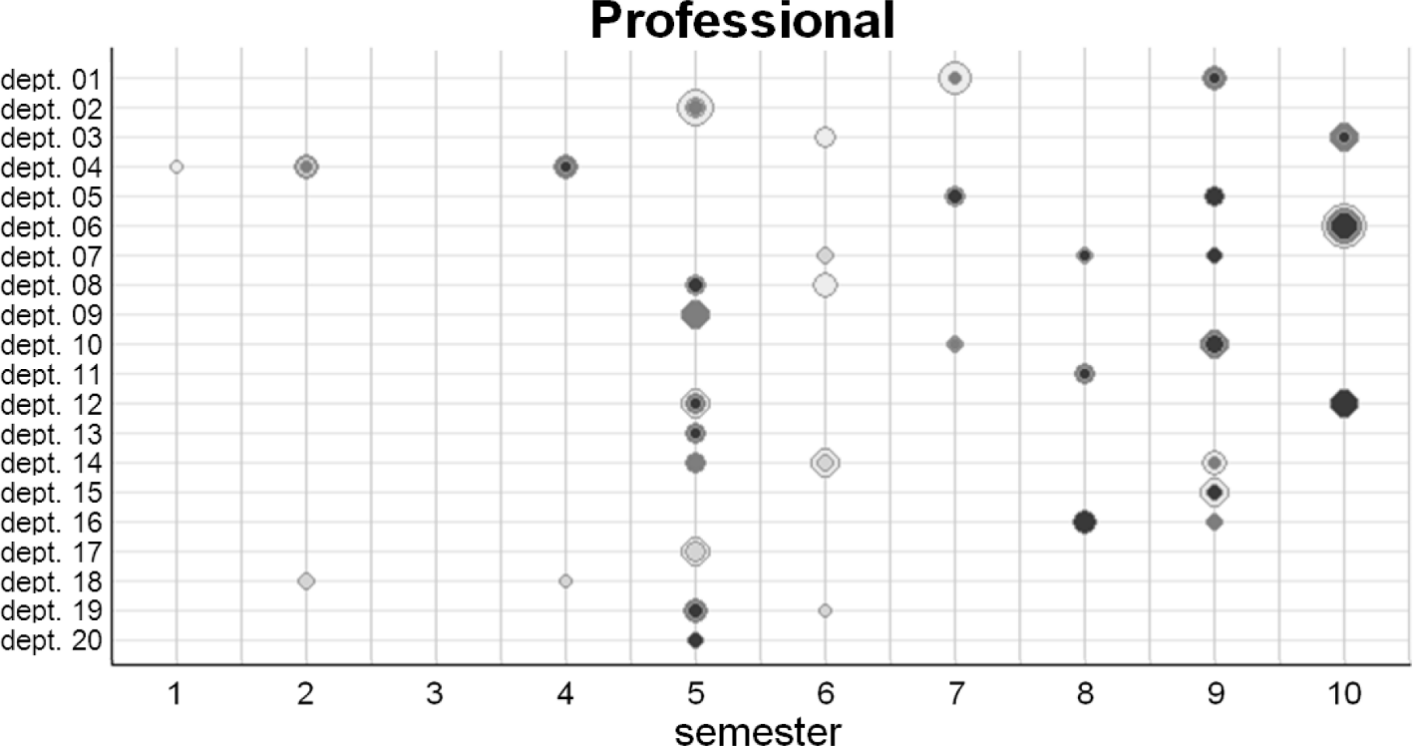
Transparency by visualising the mapping data (example): Which department (dept.) teaches which sub-competencies related with the role of the professional at what time during course of study? After entering the data into the system, several diagrams, oriented towards pragmatic issues with practical relevance from different user perspectives (e.g., professional roles’ profile, longitudinal competence development and involvement of departments, aspired levels of competence, assessment formats) are available online for the mapping coordinator. In the exemplary diagram the size of the bubbles are proportional to the number of sub-competencies taught. Various shades of grey reflect the levels of competence: the darker, the higher the level of competence.

**Benefits. **Convincing responses to the frequently posed question of sense were supplied by the MER*LIN* database [5]; cf. Part I]. With the database, the curriculum becomes transparent, the disciplines can see their involvement in teaching over the entire curriculum (“My position in the field”). Interdisciplinary communication for comparing their teaching contents and contexts is being encouraged. The automated graphs provide a visual view of the mapped data with respect to typical practical questions from various user perspectives (example in figure 1 (Fig. 1)). The common types of graphs have been quickly captured by lecturers, so that interest was stirred in further analyses of the mapping results and subsequent measures (“door opener”).

**Figure 2:**
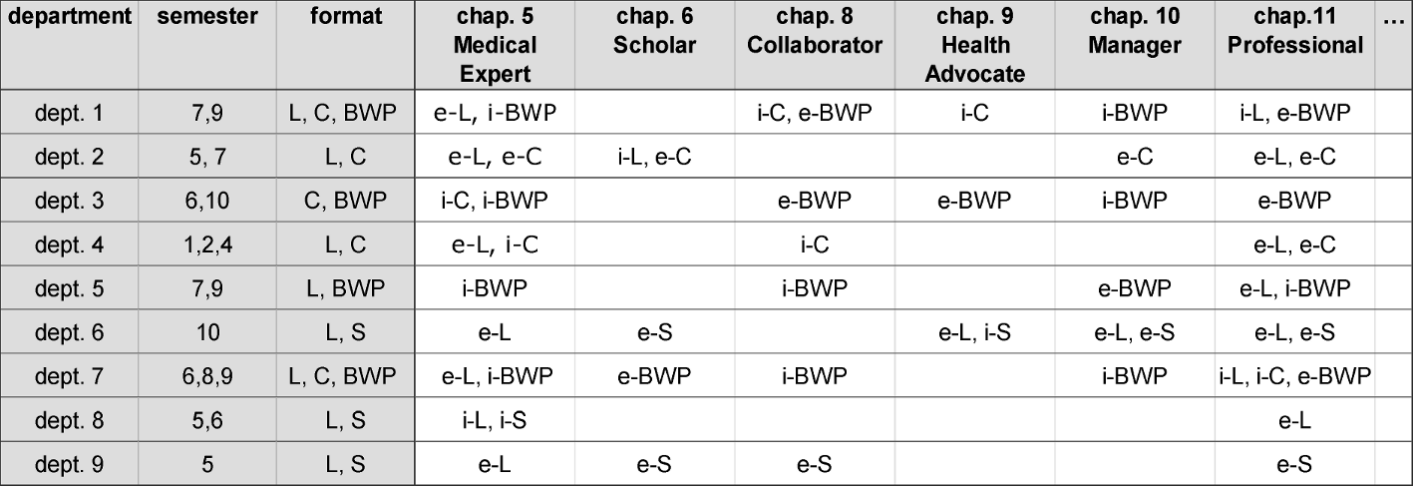
Exemplary blueprint of the preliminary students’ mapping (extract). Students mapped the individual departments courses of a faculty with respect to competence-oriented teaching ahead of the faculty’s mapping to provide support and orientation. Transfer agents/coordinators and teaching coordinators used the students’ information as an overview of which NKLM contents are primarily taught in which courses (Abbreviations: dept.=department, chap.=chapter, i=implicit, e=explicit (written form), S=seminar, L=lecture, BWP=block work placement, C=course).

**Feasibility. **The question of feasibility of curriculum mapping was of core importance and was discussed a lot at all levels, mainly because of the additional load. The following practically relevant measures have proved themselves:

1. **Substantive, technical and personal support** (cf. Part I) to meet agreed deadlines during the mapping procedure (mapping with the MERLIN database, prepared accompanying materials, accessible support from coordination and staff from the dean’s office of student affairs, quick comprehensibility of data, etc.).
2. **Portioning the NKLM** by dividing the catalogue into manageable chapters and mapping with time limits (e.g.: Chapters 5 to 11 and Chapter 14, Paragraphs a to c during the first mapping round). That approach led to a temporary reduction in the quantity of data and required effort, as well as to easier planning and early, intermediate results. Consistent monitoring by the coordinator with close -knit contact with the departments was important as guidance. Some disciplines with previous experience decided to organise the process self-directed and to go through the entire NKLM in a single step without guidance.
3. **Condensation through preliminary mapping (mapping plan/blueprint).** A successful and psychologically effective idea was the orienting “preliminary mapping” by students based on their own course experiences or accompanying materials. At the four locations, adapted variants were applied respectively. “Rough preliminary mapping” by students has proved itself: they highlighted which NKLM contents turned up in which course. That yielded a mapping plan or blueprint (see figure 2 (Fig. 2)). With these measures, the NKLM was “condensed” to the extent that lecturers no longer had to go through the entire catalogue. Focused by the blueprint, they headed directly for NKLM content and overlooked less. The other chapters were mostly skimmed through by the lecturers. In that approach, it is important to document the basis of the mapping and the procedure accurately. It is advisable, that the responsible teaching coordinator and the dean’s office of student affairs carry out a validation of the mapping data.

**Justified effort.** The contents of the newly defined professional doctors’ roles (Chapter 5 to 11) required more reflection and explanation, because of their conceptuality and novelty. They hold the danger of misunderstandings, because their contents cannot always be grasped intuitively [7]. At this point, the biggest resistance of the lecturers was observed, even though they classified the roles as relevant to practice. The expenditure of time for web-based, role-related mappings of a course of two weekly semester hours (orientating unit) was estimated at one to two hours, if guided explanatory by the coordinator. It appears that all other chapters can be mapped more easily (Chapter 14 a-c “Skills” and all chapters from the subsequent second mapping round): They are more familiar to the lecturers and decisions on the individual sub-competencies and learning objectives at NKLM Levels 2 and 3 are taken more quickly. For the more voluminous chapters too, the lecturers, being accustomed to the NKLM, usually needed two hours per course with two weekly semester hours, if the mapping was done based on an orienting mapping plan (blueprint) and with the MER*LIN* database via the internet.

Nevertheless, the reactions from many departments showed that the expenditure of time was still seen as (too) high for the moment. But automated evaluations of the mapping data based on specific practice-relevant questions and the representation of the results in familiar graphs, quickly made benefits and opportunities of curriculum mapping evident to the faculty and the lecturers. The visual demonstration of strengths and the showing of potentials for optimisation caused faculty and lecturers to open up to reflections and curricular changes.

## 4. Discussion

The empirical multicentre study at hand examines the conditions for effective NKLM curriculum mapping based on the example of the medical faculties in Baden-Württemberg. The aim was to identify relevant correlations and key factors that influence the process, as well as project-related challenges to individuals or the faculty. Deans (of study and teaching), administrative staff, students and lecturers differed in the way they could be motivated, in their openness towards certain arguments and consequently in procedures for motivation. The question of feasibility was of relevance to all groups, though for different reasons. Communication with all groups of persons was throughout effective and important, especially at a personal level. The present article focuses in particular on the motivation of the lecturers, because that group played a decisive role in the implementation of the mapping but at the same time offered the greatest resistance to it.

Methodically, a Grounded-Theory approach was selected, to come as close to reality and the interaction between phenomena and contexts as possible, without wishing to quantify the influencing factors [22]. A conclusive model resulted from constant comparisons and contrasting. Still, bias cannot be excluded. Examples are: perhaps, aspects have been wrongly interpreted; the four faculties provide different framework conditions; the lecturers knew that they were initially mapping based on the preliminary NKLM version of 2013. That could have influenced the results with respect to the size of their significance.

After the required resources and structures have been presented in Part I of this article, Part II is showing what has to be considered in the process, to be controlled and possibly contained early in order to get or keep the mapping going. This involves

1. factors and measures that are predictable and systematically plannable, and
2. problems and objections that often occur during the process and could usually be resolved by strategic argumentation.

It is not an easy task to map a complex, already existing curriculum and to convince a faculty of the benefits of mapping [9], [10], etc.]. Curriculum mapping includes significantly more data than customary catalogues of learning objective and exam-relevant topics provide. A faculty can easily compare its curriculum to the NKLM standard on the basis of electronic data: Cross connections between specialised knowledge and longitudinal acquisition of competencies can be visualised. Inconsistencies in content, teaching opportunities and examinations are revealed [13], [16], [17], [21]. The most important information for reaching data-supported decisions on curricular development are at hand. Lecturers, who see the data of their departments in the “purview of other disciplines”, reflect on their teaching. In that regard, it makes sense to think of curriculum mapping and curriculum development as a whole [2]. If achieved and proven aspects in the curriculum have to be gradually complemented as needed with additional longitudinal threads, many faculties follow the advice to preferably build up interdisciplinary competencies in longitudinally designed courses and not as course blocks [20].

Without a differentiated overview of the existing overall curriculum, the risk when integrating so-called core curricula increases significantly, that decentralized curricula evolve resp. are reinforced [2]. Although, many learning objectives are dealt with in such curricula, specialised knowledge and competencies are distributed over many semesters and courses, without mutual correlation and often without testing [2]. Curriculum mapping is considered a suitable method of gaining transparency in the curriculum [2], [8], [18], [21], [24]. For faculties planning curriculum mapping, the findings presented here may provide some assistance. The analysis of the change process at the four locations consistently revealed a series of factors, where causal relation with successful progression of the project appears plausible [1], [6], [11], [12]. From the overall consideration of the findings, ten recommendation for coordinators can be derived (see table 1 (Tab. 1)).

The measures and derived recommendations agree well with the groundbreaking publication of Bland and colleagues [1]. Based on a well-founded meta-analysis, the authors compiled a comprehensive list of key factors, that could be relevant for successful curriculum reform. They developed six categories from those factors that are of elementary importance to the success of the change process: Leadership, cooperative climate, participation by organization members, politics, human resource development, evaluation. The explanations to those superordinate success factors provide concrete tips for practical use, which have been used at the investigated locations to a large extent. In table 2 (Tab. 2), the key characteristics as well as the major measures and the related objectives of the present study have been assigned to the six categories of Bland.

## Outlook

The mapping results show, where in the comparison to the NKLM standards (further) curricular optimisations are possible or perhaps even necessary. They are already used as fundamental data source for strategic planning. Decisions can therefore be made based on evidence. Co-determination and participation in the project will be enabled at the faculties.

To ensure that curricular restructuring, as conclusive, integrated overall concept directed on competence orientation will succeed, curriculum mapping and strategic, evidence-based curriculum planning is not sufficient. In the end, it also depends on the didactical implementation and the didactical abilities of the lecturers [1]. Medical teachers do not automatically incorporate competences and roles in their teaching [14]. For individual doctors’ roles that are altogether seen as relevant to practice, the understanding of the lecturers does clearly differ from the NKLM definitions [7]. Therefore, since 2013 competence orientation has been a leading issue in medical-didactic training in Baden-Württemberg. Lecturers can become familiar with the requisite didactic knowledge and adequate concepts and acquire qualification points as inducement when reviewing their actual courses. Through transparent communication about the curriculum, the students can also be challenged and involved more closely to their benefit. Finally, the creation of suitable framework conditions for teaching, with acknowledgement and necessary support, is crucial for success. Seen as a whole, the objective of lasting change of teaching culture must be the creation of conducive framework conditions, involving the entire faculty.

## Acknowledgements

We are grateful to the entire MER*LIN* group for the stimulating discussions. We also wish to express our thanks to Dr Wolfgang Öchsner and Mrs Claudia Grab (both of Ulm University) for their contributions during pilot mapping, as well as to the deans of study and teaching, the staff of the deans’ offices of student affairs, lecturers and students, who supported the mapping project with full engagement. Particularly, we wish to thank all teaching coordinators and those responsible for modules for their energetic participation in the mapping process and the discussions. They contributed important information and gave impulses for optimising the instruments and the process.

## Funding

The project is funded by the Federal Ministry of Education and Research (BMBF [Bundesministerium für Bildung und Forschung]) within the framework of the promotional programme “Quality Pact for Teaching” (QPL [Qualitätspakt Lehre]); the joint project MER*LIN* (*Medical Education Research, Lehrforschung im Netz* [study research within the network]) of the medical faculties of the universities of Freiburg, Heidelberg, Mannheim and Tübingen, led by Tübingen, reference number: 01Pl12011A.

## Competing interests

The authors declare that they have no competing interests.

## Figures and Tables

**Table 1:**
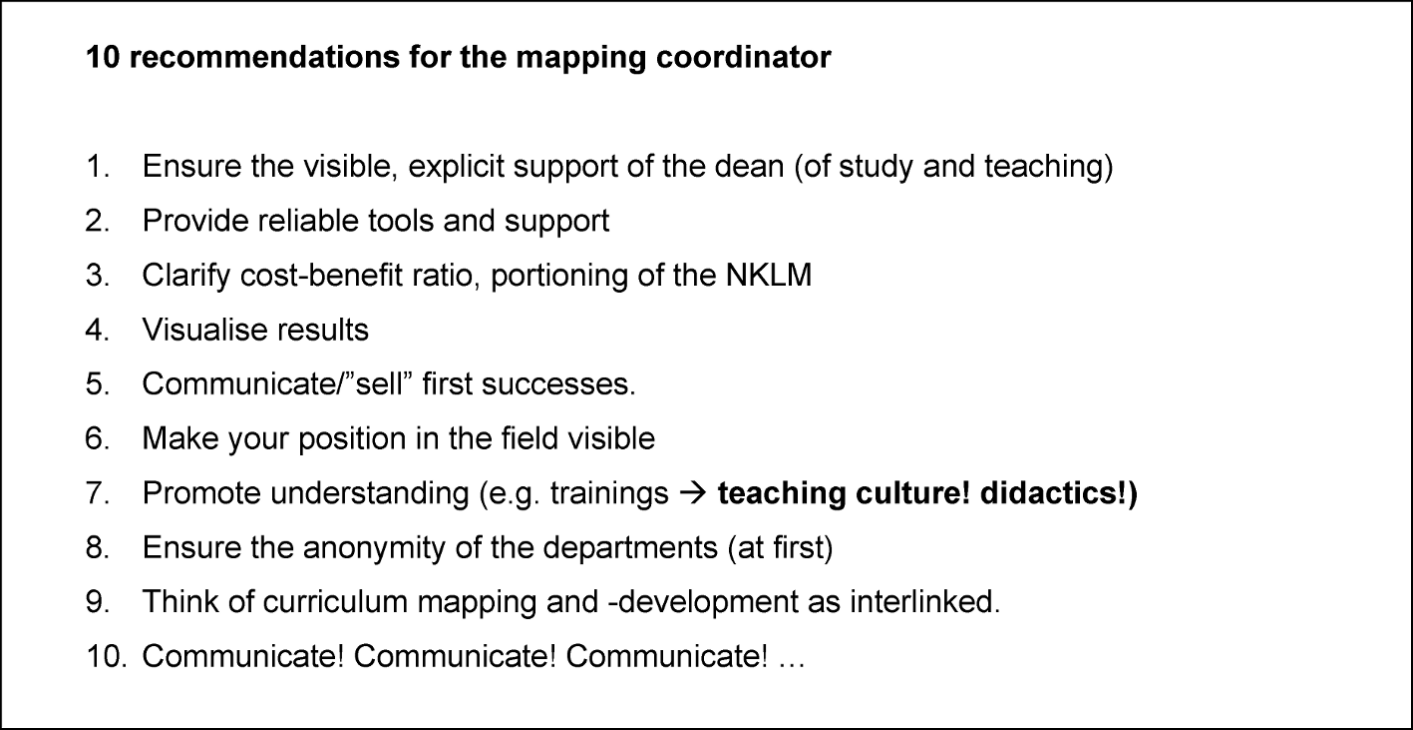
Useful tips to facilitate the NKLM mapping in the process. The recommendations were derived from experiences made during the NKLM mapping at four medical faculties in Baden-Württemberg.

**Table 2:**
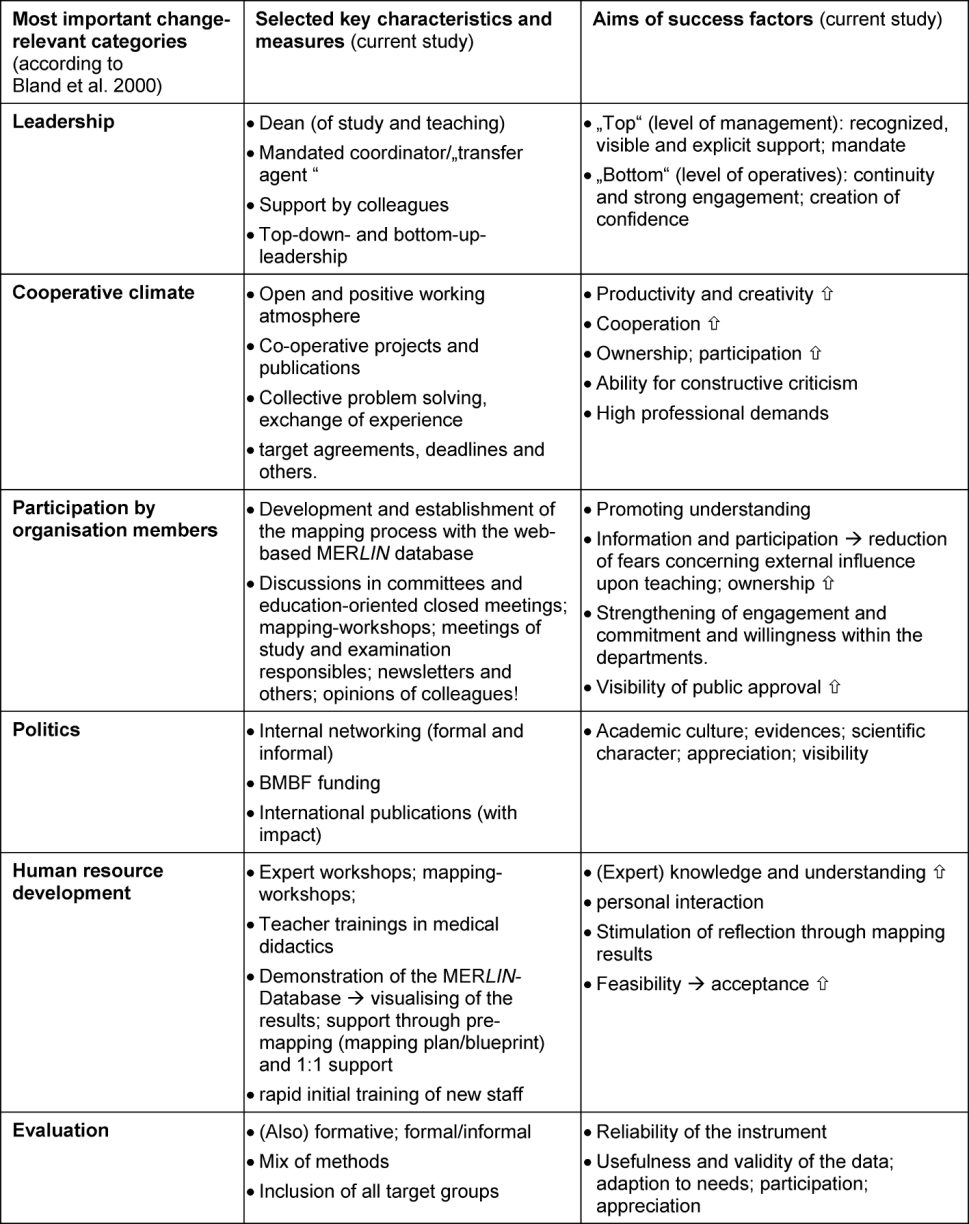
Tabular overview of the most important factors within the change process in curriculum mapping in comparison to the most important change-relevant categories to Bland et al. 2000. The conducive resources and structures that should be installed at the beginning of the project are discussed in Part I of this article; all factors and measures supporting and accompanying the process are explained in Part II.

## References

[1] Bland CJ, Starnaman S, Wersal L, Moorhead-Rosenberg L, Zonia S, Henry R (2000). Curricular Change in Medical Schools: How to Succeed. Acad Med.

[2] Davis MH, Harden RM (2003). Planning and implementing an undergraduate medical curriculum: the lessons learned. Med Teach.

[3] Fischer MR, Bauer D, Mohn K, NKLM-Projektgruppe (2015). Finally Finished! National Competence Based Catalogues of Learning Opjectives for Undergraduate Medical Education (NKLM) and Dental Education (NKLZ) ready for trial. GMS Z Med Ausbild.

[4] Frenk J, Chen L, Bhutta ZA, Cohen J, Crisp N, Evans T, Fineberg H, Garcia P, Ke Y, Kelley P, Kistnasamy B, Meleis A, Naylor D, Pablos-Mendez A, Reddy S, Scrimshaw S, Sepulveda J, Serwadda D, Zurayk H (2010). Health professionals for a new century: transforming education to strengthen health systems in an interdependent world. Lancet.

[5] Fritze O, Boecker M, Gornostayeva M, Durante S, Griewatz J, Öchsner W, Narziß E, Wosnik A, Lammerding-Köppel M (2014). Kompetenzorientiertes Curriculummapping im MERLIN-Projekt: eine Online-Datenbank als Tool zur gezielten curricularen Weiterentwicklung. http://dx.doi.org/10.3205/14gma255.

[6] Gale R, Grant J (1997). AMEE Medical Education Guide No. 10: Managing change in a medical context: Guidelines for action. Med Teach.

[7] Griewatz J, Wiechers S, Ben-Karacobanim H, Lammerding-Koeppel M (2016). Medical teachers' perception of professional roles in the framework of the German National Competence-Based Learning Objectives for Undergraduate Medical Education (NKLM) – a multi-centre study. Med Teach.

[8] Harden RM (2001). AMEE guide no. 21: Curriculum mapping: A tool for transparent and authentic teaching and learning. Med Teach.

[9] Holycross, J (2006). Curriculum mapping--an essential tool for curriculum development. Am J Pharm Educ.

[10] Kelley KA, McAuley JW, Wallace LJ, Frank SG (2008). Curricular Mapping: Process and Product. Am J Pharm Educ.

[11] Lane IF (2007). Change in higher education: Understanding and Responding to individual and organizational resistence. J Vet Med Educ.

[12] Loeser H, O'Sullivan P, Irby DM (2007). Leadership Lessons from Curricular Change at the University of California, San Francisco School of Medicine. Acad Med.

[13] Plaza, CM, Reierson Draugalis, J, Slack MK, Skrepnek, GH, Sauer KA (2007). Curriculum mapping in program assessment and evaluation. Am J Pharm Educ.

[14] Renting N, Dornan T, Gans RO, Borleffs JC, Cohen-Schotanus J, Jaarsma AD (2016). What supervisors say in their Feedback: construction of CanMEDS roles in workplace settings. Adv Health Sci Educ Theory Pract.

[15] Robley W, Whittle S, Murdoch-Eaton D (2005). Mapping generic skills curricula: a recommended methodology. J Further Higher Educ.

[16] Robley W, Whittle S, Murdoch-Eaton D (2005). Mapping generic skills curricula: outcomes and discussion. J Further Higher Educ.

[17] Smith SR, Dollase R (1999). AMEE guide no. 14: Planning, implementing and evaluating a competency-based curriculum. Med Teach.

[18] Tractenberg RE, Umans JG, McCarter RJ (2010). A mastery rubric: Guiding curriculum design, admissions and development of course objectives. Assess Eval High Educ.

[19] Uchiyama KP, Radin JL (2009). Curriculum mapping in higher education: A vehicle for collaboration. Innovat High Educ.

[20] Van Dalen J, Kerkhofs E, van Knippenberg-Van Den Berg BW, van Den Hout HA, Scherpbier AJJA, van Der Vleuten CPM (2002). Longitudinal and concentrated communication skills programmes: two dutch medical schools compared. Adv Health Sci Educ.

[21] Veltri NF, Webb HW, Matveev AG, Zapatero EG (2011). Curriculum mapping as a tool for continuous improvement of IS curriculum. JISE.

[22] Watling CJ, Lingard L (2012). Grounded theory in medical education research: AMEE Guide No.70. Med Teach.

[23] Wissenschaftsrat (WR) (2014). Empfehlungen zur Weiterentwicklung des Medizinstudiums in Deutschland auf Grundlage einer Bestandsaufnahme der humanmedizinischen Modellstudiengänge.

[24] Zelenitsky S, Vercaigne L, Davies NM, Davis C, Renaud R, Kristjanson C (2014). Using Curriculum Mapping to Engage Faculty Members in the Analysis of a Pharmacy Program. Am J Pharm Educ.

